# Association of hospital centrality in inter-hospital patient-sharing networks with patient mortality and length of stay

**DOI:** 10.1371/journal.pone.0281871

**Published:** 2023-03-15

**Authors:** Regan W. Bergmark, Ginger Jin, Robert S. Semco, Marc Santolini, Margaret A. Olsen, Amar Dhand

**Affiliations:** 1 Center for Surgery and Public Health, Brigham and Women’s Hospital and Harvard Medical School, Boston, MA, United States of America; 2 Brigham and Women’s Hospital and Dana Farber Cancer Institute and Department of Otolaryngology-Head and Neck Surgery, Division of Otolaryngology-Head and Neck Surgery, Harvard Medical School, Boston, MA, United States of America; 3 Université Paris Cité, Inserm, System Engineering and Evolution Dynamics, Paris, France; 4 Network Science Institute, Northeastern University, Boston, MA, United States of America; 5 Department of Medicine, Division of Infectious Disease, Washington University School of Medicine, St. Louis, MO, United States of America; 6 Department of Neurology, Brigham and Women’s Hospital, Harvard Medical School, Boston, MA, United States of America; Albert Einstein College of Medicine, UNITED STATES

## Abstract

**Objective:**

The interdependence of hospitals is underappreciated in patient outcomes studies. We used a network science approach to foreground this interdependence. Specifically, within two large state-based interhospital networks, we examined the relationship of a hospital’s network position with in-hospital mortality and length of stay.

**Methods:**

We constructed interhospital network graphs using data from the Healthcare Cost and Utilization Project and the American Hospital Association Annual Survey for Florida (2014) and California (2011). The exposure of interest was hospital centrality, defined as weighted degree (sum of all ties to a given hospital from other hospitals). The outcomes were in-hospital mortality and length of stay with sub-analyses for four acute medical conditions: pneumonia, heart failure, ischemic stroke, myocardial infarction. We compared outcomes for each quartile of hospital centrality relative to the most central quartile (Q4), independent of patient- and hospital-level characteristics, in this retrospective cross-sectional study.

**Results:**

The inpatient cohorts had 1,246,169 patients in Florida and 1,415,728 in California. Compared to Florida’s central hospitals which had an overall mortality 1.60%, peripheral hospitals had higher in-hospital mortality (1.97%, adjusted OR (95%CI): Q1 1.61 (1.37, 1.89), p<0.001). Hospitals in the middle quartiles had lower in-hospital mortality compared to central hospitals (%, adjusted OR (95% CI): Q2 1.39%, 0.79 (0.70, 0.89), p<0.001; Q3 1.33%, 0.78 (0.70, 0.87), p<0.001). Peripheral hospitals had longer lengths of stay (adjusted incidence rate ratio (95% CI): Q1 2.47 (2.44, 2.50), p<0.001). These findings were replicated in California, and in patients with heart failure and pneumonia in Florida. These results show a u-shaped distribution of outcomes based on hospital network centrality quartile.

**Conclusions:**

The position of hospitals within an inter-hospital network is associated with patient outcomes. Specifically, hospitals located in the peripheral or central positions may be most vulnerable to diminished quality outcomes due to the network. Results should be replicated with deeper clinical data.

## Introduction

The interdependence of hospitals is underappreciated in contemporary patient outcomes studies. Hospital and patient outcomes are not determined only by the quality of care delivered on site, but also by the quality of care provided at other hospitals connected to the index site. For example, poor infection control at one hospital can lead to higher infection rates at another hospital via patient transfers [1]. Such hospital-to-hospital connections may be defined indirectly through geographic proximity, or directly by measuring the movement of patients from one hospital to another. Such patient movement across facilities may be due to physician-to-physician referrals, hospital transfers, or simply patient choice. Hospitals can therefore be studied as embedded in complex networks of facilities that are interconnected and interdependent. This wider ecological perspective has important implications for interpreting hospital-level outcomes and patient outcomes. Mapping the network may also enable new approaches to quality improvement interventions.

Hospitals may have interdependent structures and processes that affect patient outcomes. The abilities to provide care close to a patient’s home, transfer patients to a higher level of care if needed, or directly provide high quality complex care demonstrate hospital and health system interdependence. Researchers have demonstrated the interdependence of hospitals by showing that outcomes are linked across facilities. For example, there is strong evidence for the regional dissemination of hospital-acquired infections [1–4]. In particular, researchers found that MRSA outbreaks rapidly spread within networks defined based on shared patients [1–3]. In fact, the proportion of shared patients between hospitals was correlated with genetic similarity of MRSA strains from those hospitals [4]. Clostridium difficile infections are likewise shown to be associated with a hospital’s network connectivity based on transferred patients [5,6]. There is also differential quality of care effects of inter-hospital critical care transfers, with transferred patients more like to go to higher-quality centers [7,8] and hub-and-spoke networks for stroke and myocardial infarction with reliance on multiple sites of care [9,10]. In critical care transfers, the centrality of a hospital is associated with increased capability in delivery of services [7]. Although well resourced, such hospital hubs may come under strain during healthcare crises such as the COVID-19 pandemic or natural disasters that rely on hubs to manage surges of patients. Patient outcomes could be affected due to this positional strain. Therefore, analysis of patient-focused healthcare utilization networks such as these allows researchers to investigate how patients move through the health care system and subsequently whether their outcomes are influenced by the hospital network around them.

Hospital quality improvement efforts and quality and value measurements may fail to include these network effects despite increasing hospital collaboration and mutual reliance. Most obviously, an acceleration of mergers and acquisitions have led to increases in formal financial ties between hospitals. Hospital participation in regional networks has doubled in the last decade [11]. Patient-sharing and inter-dependence also occurs between financially independent hospitals. Yet, most current hospital quality measurements emphasize the importance of accounting for differences in patient case mix [12] and hospital characteristics but not inter-hospital network features [13]. Hospital performance metrics for example rankings are based on hospital-acquired infections [14], thrombolysis rates [15], and other metrics highly dependent on network position and patient flow. Network metrics may highlight the importance of implementing patient-sharing protocols that optimize resilience and performance of the whole ecosystem. A network approach could benefit the system at large by improving patient outcomes and costs.

In this paper, we examine a method to incorporate hospital network structure into the risk adjustment of patient mortality and length of stay (LOS). Our objective is to determine whether a hospital’s centrality in an inter-hospital network was associated with inpatient mortality and LOS in 2 states, Florida and California using state inpatient databases. We hypothesized that there would be clinically significant associations between hospital network centrality and patient outcomes after controlling for typical patient and hospital factors.

## Methods

In a retrospective cross-sectional study, we conducted a network analysis of hospitals that shared patients in Florida and California. To do this, we used an administrative claims dataset to build the networks, defined the network characteristics of the full network and each individual hospital, and then examined the association of hospital network characteristics with hospital quality outcomes.

### Data source

We used administrative claims data from the State Inpatient Database (SID) [16] and State Emergency Department Database (SEDD) [17] from the Healthcare Cost and Utilization Project (HCUP) for network creation and SID alone for patient outcomes in this retrospective cross-sectional study. HCUP is the largest collection of multiyear, all-payer discharge data in the United States and includes state-level datasets from participating states. The SID “contain[s] all inpatient care records in participating states…more than 95 percent of all U.S. hospital discharges [18].” Specific states such as Florida and California include an encrypted identifier (visitlink variable) to link encounters longitudinally. These states were chosen because of this longitudinal identifier and their large sizes. The SEDD includes all state-specific treat-and-release hospital emergency department patient visits (ED visits resulting in admission are included in the SID). SID contains all state-specific inpatient patient visits to acute care community hospitals. We used the 2014 SEDD and SID from Florida (January 1-December 31, 2014). We validated the analysis by using the 2011 SEDD and SID from California (January 1-December 31, 2011), the most recent year available through HCUP at the time of this study. We chose the two states because of their size, diversity of demographics, and the limited movement of patients for care out of state. Additionally, these two state datasets have unique patient identifiers and timing indicators, variables called ‘visitlink’ and ‘daystoevent’, allowing tracking of individuals longitudinally within a state. Additional information on hospital characteristics were obtained from the American Hospital Association annual survey (AHA) files from 2011–2012 [19].

### Hospital network creation

We built the networks for each state using all adult emergency and inpatient visits (SEDD and SID) for the given year regardless of diagnosis, to ensure the network was comprehensive and to limit the introduction of potential bias. We used both types of visits because we wanted to broadly define the network of patient-sharing within a state. Inclusion of both allowed us to include patients with a variety of illness severity, rather than only patients who require inpatient treatment at different facilities. The *network nodes* were all non-federal acute care hospitals in each state. The *network links* were the number of patients who were seen in the emergency department or inpatient service of one hospital and then the emergency department or inpatient service of another hospital at any time during the study period. Links were treated as undirected and included all movement between hospitals including hospital transfers, physician-referred care, and patient-determined care. Each state was analyzed separately. The SID data does not include encounters from other states, and therefore all data only reflect healthcare use within the state, excluding the contribution of out-of-state hospitals on the local network structure.

**Fig 1:**
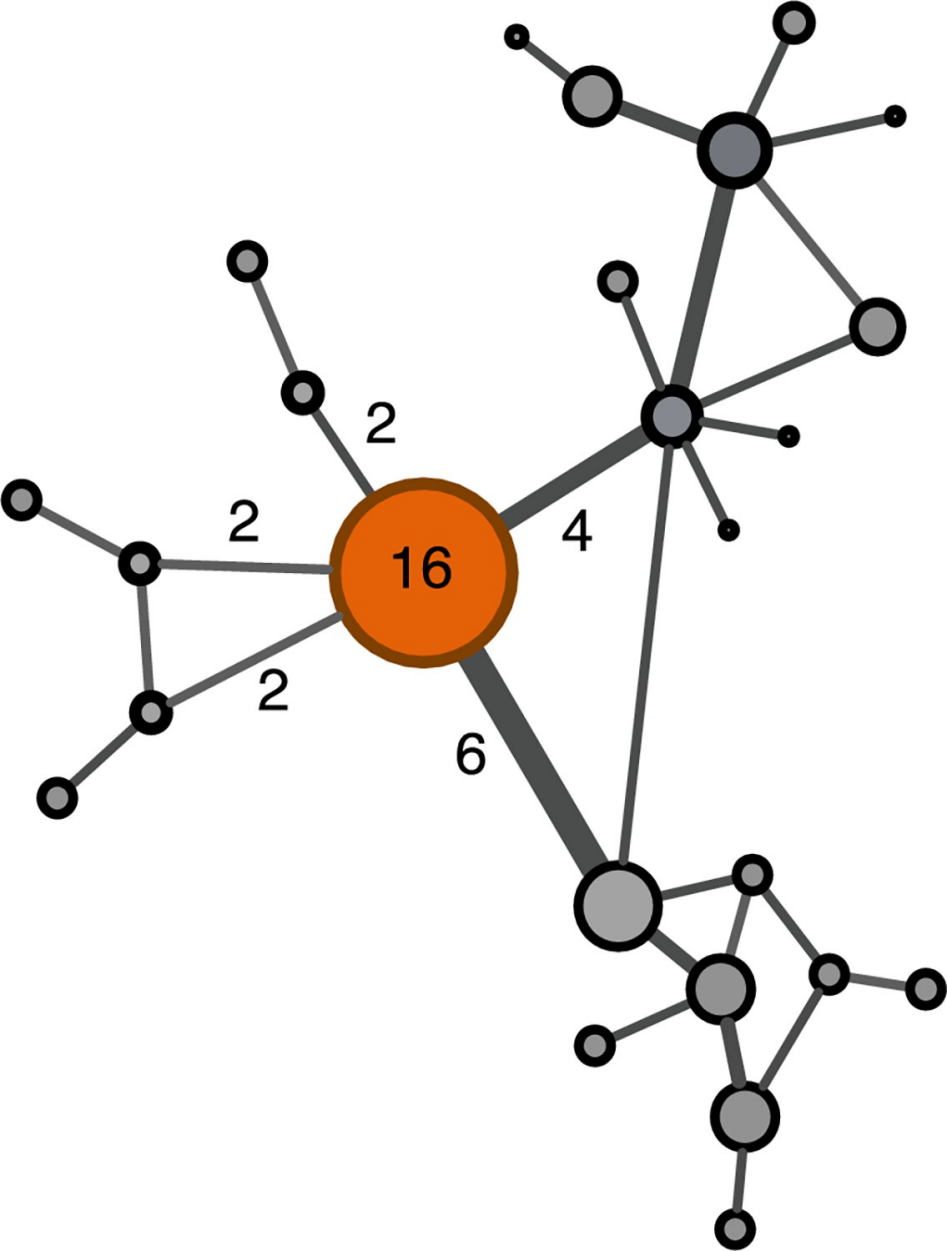
Example of weighted degree. The weighted degree, 16, of the central hospital is the sum of all patients shared with other hospitals (4+6+2+2+2).

To build the network, we began by creating an adjacency network in which every hospital was listed as a row and also as a column. A numerical value was assigned equivalent to the number of patients shared by each of two hospitals. The R program *igraph* was used to calculate global network characteristics including weighted degree as the centrality measure. Weighted degree is the sum of all weighted connections to a given hospital in the network (Fig 1) [20]. Weighted degree increases as the number of its ties to other hospitals increases, meaning the number of other hospitals with which patients are shared. It also increases as the strength of each tie increases, meaning the number of shared patients with each hospital [21]. For analysis, we used the calculated weighted degree for each hospital without transformation. For visualization, we extracted the multiscale backbone of the weighted network as described in Serrano et al. (2009) [22].

### Study population

For the analysis of outcomes, we included all patients ≥ 18 years old who had at least one hospital discharge from the SID file during the study time for each specific state (Florida: 2014; California: 2011). Patients with multiple discharges were counted once (the first visit) in line with standard hospital outcome studies. This approach avoided co-linearity of patient data for patients with multiple discharges and removed the need to censor patients with an initial outcome of death. We completed analysis on the overall cohort and four acute medical condition-specific subgroups: heart failure, myocardial infarction, or pneumonia [23] or ischemic stroke [24]. These conditions were chosen through review of the most frequent in-hospital mortality diagnoses in the US, choosing the top four with validated diagnostic codes. International Classification of Diseases, Ninth Edition, Clinical Modification (ICD-9-CM) diagnosis codes were used that have been previously validated (S6 Appendix). This disease-specific approach was taken to further examine the impact of hospital network characteristics among patients with specific medical conditions. Our study was approved by the Institutional Review Board at Partners Healthcare, now Mass General Brigham (Protocol #2015P001722).

### Exposure & outcomes

The main exposure variable was facility weighted degree quartile. All facilities were divided into four quartiles according to their weighted degree, where the first quartile contained hospitals that were most peripheral in the network and the fourth quartile contained hospitals that were most central.

The main outcome variables were measures of hospital quality including: 1) inpatient mortality, defined as death that occurred in the hospital, and 2) mean hospital length of stay (LOS), defined as the mean time between admission and discharge.

### Covariates

We adjusted for the patient-level and hospital-level covariates in our multi-level analysis with patient-level variables in our first level of hospital level variables in the second level. Patient-level covariates were age (years), gender, race (Non-Hispanic White, non-Hispanic Black, Hispanic, Other), median household income by quartile, payers (Medicare, private, Medicaid, self-pay, other; other could include other sources of coverage or having no charge levied), patient location (Large metropolitan area, small metropolitan area, micropolitan area), and Charlson comorbidity index (CCI) (0, 1, 2, 3, >3) [25]. Hospital-level covariates were control ownership of hospital (private/not-for-profit, government/nonfederal, investor-owned for-profit), hospital teaching status (yes/no), and hospital bed-size (1–99 beds, 100–199 beds, 200–299 beds, > = 300 beds). For the California cohort only, we also adjusted for patient admission source (Emergency, routine including referral and outpatient, transfer, court/law enforcement). Missing data were reported in the patient characteristics (for example, missing insurance data).

### Statistical analyses

We conducted a multilevel logistic regression to determine the association of hospital centrality and inpatient mortality in the 2014 Florida cohort. A multilevel model helps to account for this hierarchical structure. The reference category was the most central quartile (Q4) as we expected this quartile to have the largest number of admissions. We used multilevel logistic regression analyses to account for hospital-level and patient-level factors. Specifically, hospital clustering was accounted for using a random-effect model. We used negative binomial regressions to determine the association of hospital centrality with LOS because this model better fits the data after adjusting for all potential risk factors. We calculated the incidence rate ratio (IRR) for length of stay by hospital quartile relative to the most central quartile. For mortality, we calculated adjusted odds ratios. For validation, we replicated these analyses in the California cohort and for the previously defined disease-specific cohorts (ischemic stroke, heart failure, myocardial infarction, and pneumonia).

In sensitivity analyses, we analyzed LOS among those patients who did not die in hospital to minimize the effect that early in-hospital death has on the shortening of length of stay. We described categorical variables using frequencies and percentages and we described continuous measures using means and standard deviations. We used a Pearson chi-square test and a 2-sided robust t-test to determine the unadjusted differences as appropriate. We used a P-value of 0.05 as the threshold for significance. We used R 3.5.0 (R Core Team, 2018) to build the network and SAS (version 9.4, SAS Institute Inc., Cary, NC) to complete the multilevel analyses.

## Results

### Network characteristics

**Fig 2:**
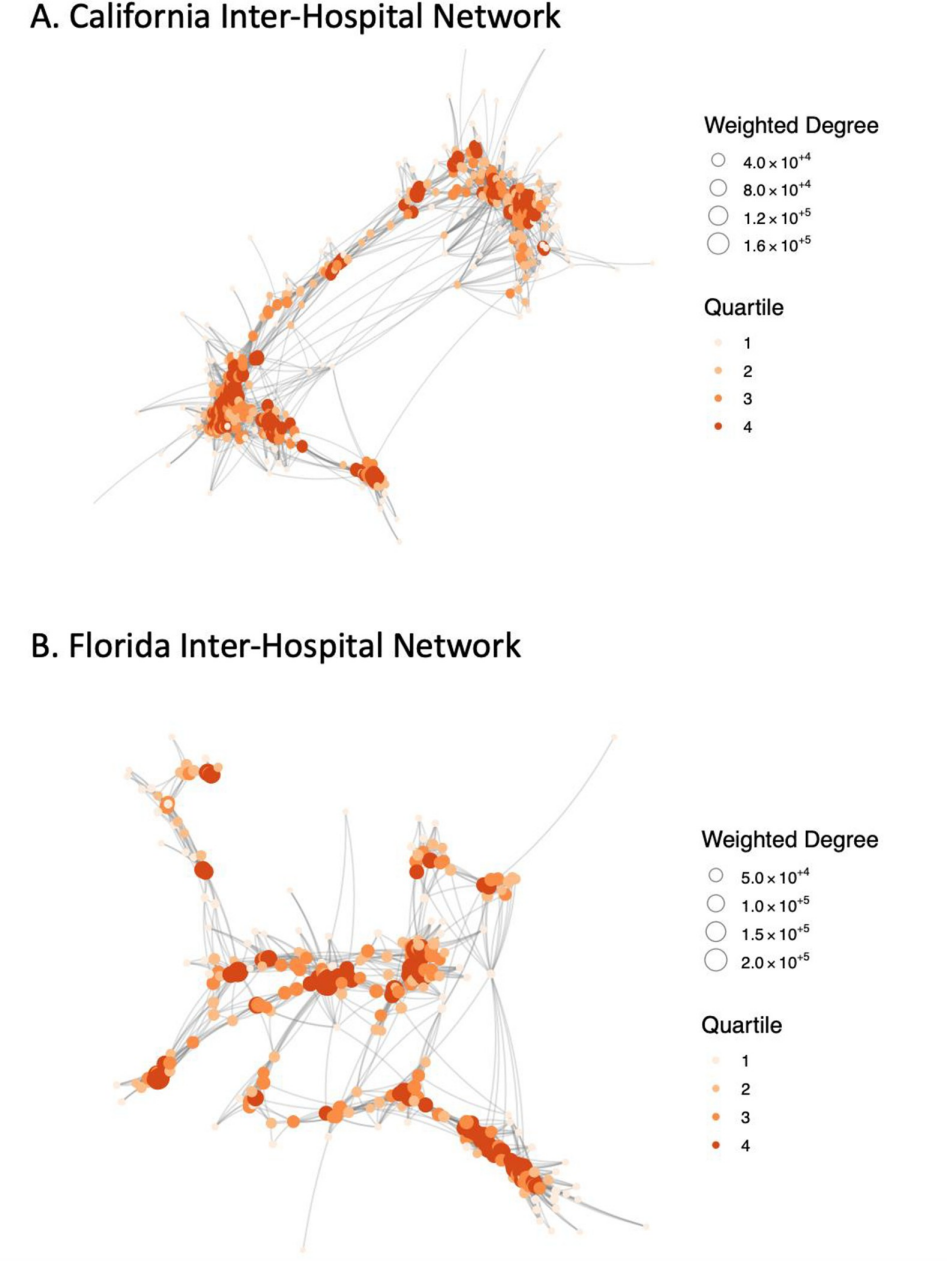
Inter–hospital networks in A) California 2011 and B) Florida 2014, where each node represents one hospital, and the links represents shared patients. Hospital node size represents the hospital’s weighted degree, where larger nodes have higher degrees and are more central in the network. Hospital node color represents the hospital’s centrality quartile, where the dark red nodes (quartile 4) are most central. Hospitals are clustered according to how many patients they share.

**Fig 3:**
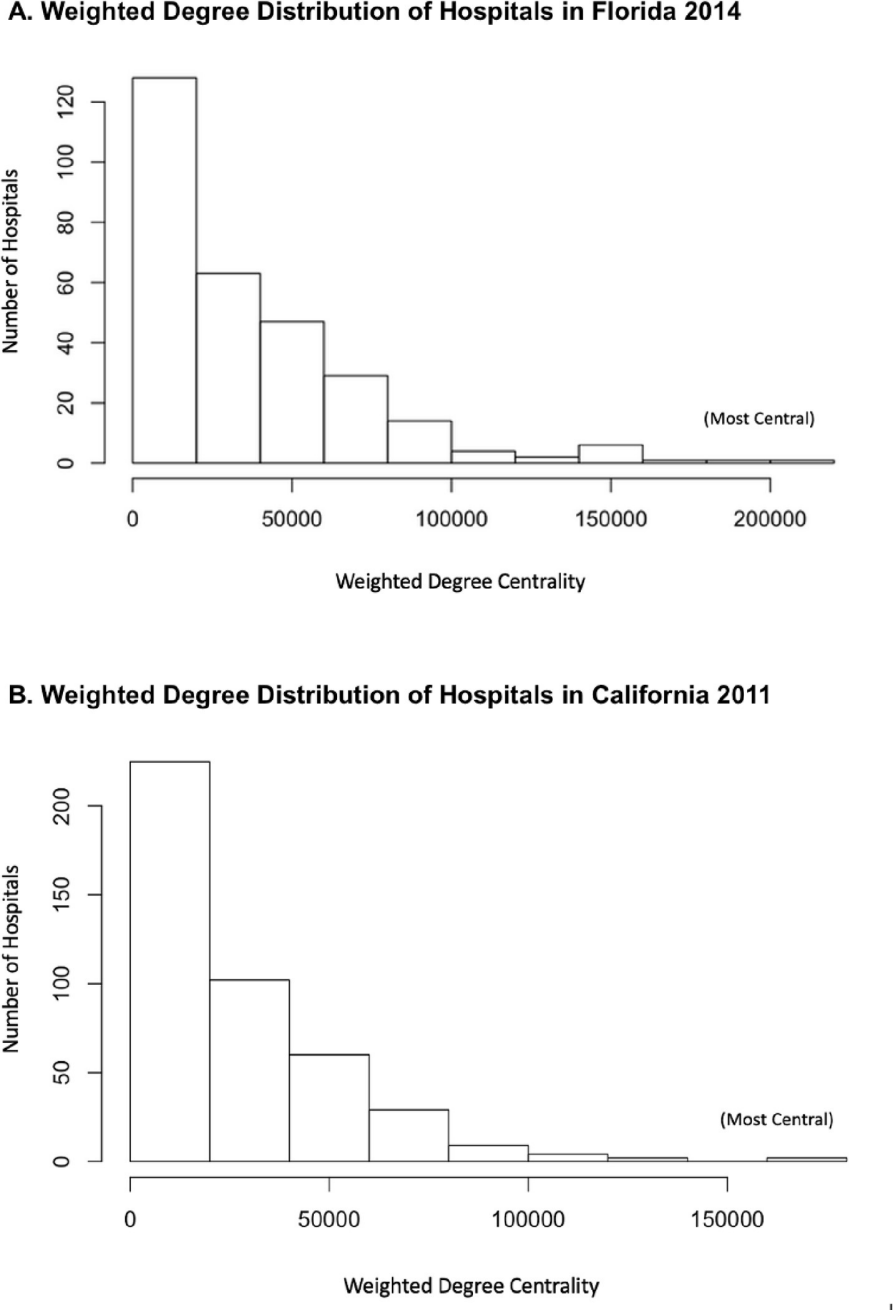
Distribution of weighted degree centrality of hospitals in A) Florida and B) California. These histograms show a high number of low centrality (peripheral) hospitals and a low number of high frequency (hub) hospitals.

Fig 2 shows the hospital networks for Florida and California with nodes sizes according to weighted degree and color according to quartile. The inter-hospital network for Florida in 2014 consisted of 296 inpatient facilities and 25,364 links, representing connections between facilities due to patient-sharing. The density of the network was 0.58, meaning that out of all possible links that could exist between the set of facilities, 58% of them existed in this network. The global transitivity for the network was 0.78, indicating that there is a high degree of clustering between nodes. In other words, 78% of the immediately connected hospitals of a specific hospital are also linked together. As is characteristic of many real networks, there was a minority of hospitals with a high centrality and a majority of hospitals with a low centrality, approximated well by a negative binomial distribution (Fig 3A).

The inter-hospital network for California in 2011 consisted of 433 nodes and 46659 links. The density of the network in California was 0.50, and the global transitivity for the network was 0.69. Therefore, the California hospital network had lower connectivity and clustering than Florida. On the other hand, similar to the Florida network, there was a minority of hospitals with a high centrality and a majority of hospitals with a low centrality, approximated well by a negative binomial distribution (Fig 3B).

As described in the methods, the hospitals were ranked according to their centrality score and categorized into quartiles. The number of unique patients per hospital was heavily skewed towards more central hospitals, as the hospitals in the most central quartile provided care to 51% of all patients in the study period, while the hospitals in the most peripheral quartile provided care to only 2.4% of all patients.

### Patient level variables

In Florida, there were 1,246,169 discharges with 18,672 (1.50%) in-hospital deaths. The mean age of patients was 62.7 years (standard deviation [SD] 18.6), and 52.8% were female. Of the patient population, 69.2% were non-Hispanic White, 14.0% were non-Hispanic Black,14.4% were Hispanic, and 2.5% were other races or ethnicities. Medicare was the primary insurer for 56.3% of patients, while 21.9% had private insurance, 9.3% had Medicaid, and 9.1% were self-pay or other/received no charge. As defined by the Charlson Comorbidity Index, 39.8% of patients had no comorbidities, while 20.5% had 3 or greater. 60.1% of patients lived in large metropolitan areas, 35.4% in small metropolitan areas, and 4.5% in micropolitan areas.

In California, there were 1,415,728 discharges with 30,617 (2.16%) in-hospital deaths. The mean age of patients was 62.0 years (SD 18.4) and 54.3% of patients were females. The study population consisted of 60.9% non-Hispanic white patients, 8.4% non-Hispanic black patients, and 21.1% Hispanic patients. Nearly half of all patients had coverage through Medicare (49%), while 29% were covered through private insurance, 11.9% through Medicaid, and 10.5% were self-pay or other/received no charge. As defined by the Charlson Comorbidity Index, 40% of patients had no comorbidities, while 22% had 3 or greater. The large majority of patients were from metropolitan areas, with 75.3% from large metropolitan areas, 21.8% from small metropolitan areas, and 2.9% from micropolitan areas. See Table 1 for summary of the patient populations from Florida and California.

**Table 1:** Patient characteristics in Florida 2014 and California 2011.

	Florida		California	
	N	%	N	%
**Hospitalizations, No (%)**	1,246,169	100	1,415,728	100
**Age (mean ± SD)**	62.7 ± 18.6		62.0 ± 18.4	
**Age (yr)**				
18–39 No (%)	162,347	13.0	182,480	12.9
40–49 No (%)	132,585	10.6	174,732	12.3
50–59 No (%)	206,336	16.6	252,026	17.8
60–69 No (%)	241,841	19.4	266,628	18.8
70–79 No (%)	246,045	19.7	246,194	17.4
80+ No (%)	257,015	20.6	293,668	20.7
**Gender**				
Male	588,132	47.2	647,499	45.7
Female	658,037	52.8	768,229	54.3
**Race**				
Non-Hispanic White	861,706	69.2	862,794	60.9
Non-Hispanic Black	174,311	14.0	119,100	8.4
Hispanic	178,893	14.4	298,981	21.1
Other	31,259	2.5	134,853	9.5
**Median household income**				
Low (Quartile 1)	359,750	28.9	370,144	26.1
Moderate (Quartile 2)	334,233	26.8	354,022	25.0
High (Quartile 3)	304,640	24.5	367,375	25.9
Very high (Quartile 4)	247,546	19.9	324,187	22.9
**Patient location**				
Large metro	749,396	60.1	1,066,023	75.3
Small metro	440,459	35.4	308,171	21.8
Micropolitan	56,314	4.5	41,534	2.9
**Payers**				
Medicare	701,208	56.3	693,097	49.0
Private Insurance	273,150	21.9	405,810	28.7
Medicaid	115,317	9.3	168,787	11.9
Self-pay	88,337	7.1	64,515	4.6
No charge	24,760	2.0	--	--
Other/Missing	43,397	3.5	83,519	5.9
**CCI**				
0	496,414	39.8	562,895	39.8
1	306,957	24.6	332,729	23.5
2	186,346	15.0	209,478	14.8
3	103,540	8.3	112,466	7.9
>3	152,912	12.3	198,160	14.0
In-Hospital Mortality	18,672	1.50	30,617	2.16

### Hospital level variables

In Florida, the patient population was admitted to 272 different hospitals over the course of the study period. Of these, 23 (8.5%) were major teaching hospitals, 58 (21.3%) were minor teaching hospitals, and 191 (70.2%) were non-teaching hospitals. 56% of hospitals had fewer than 200 beds, while 29% had greater than 300 beds (S1 Appendix). When examined by hospital network centrality quartile, most hospitalizations (N = 691,616, 55.5%) occurred in hospitals in the most central quartile (quartile 4), followed by quartile 3 (N = 373,563, 30.0%), and quartile 2 (N = 161,028, 13.2%). The fewest hospitalizations (N = 16,962, 1.4%) occurred in the least central quartile (S2 Appendix).

In California, the patient population was admitted to 396 different hospitals over the course of this study period. Of these, 20 (5.1%) were major teaching hospitals, 77 (19.4%) were minor teaching hospitals, and 299 (75.5%) were non-teaching hospitals. 60% of hospitals had fewer than 200 beds, while 24% had greater than 300 beds (S1 Appendix). Similar to Florida, when examined by hospital network centrality quartile, most hospitalizations (N = 747,313, 52.8%) occurred in hospitals in the most central quartile, whereas the fewest (N = 35,505, 2.5%), occurred in the least central quartile (S3 Appendix).

### Hospital centrality quartile and inpatient mortality and mean length of stay

**Fig 4:**
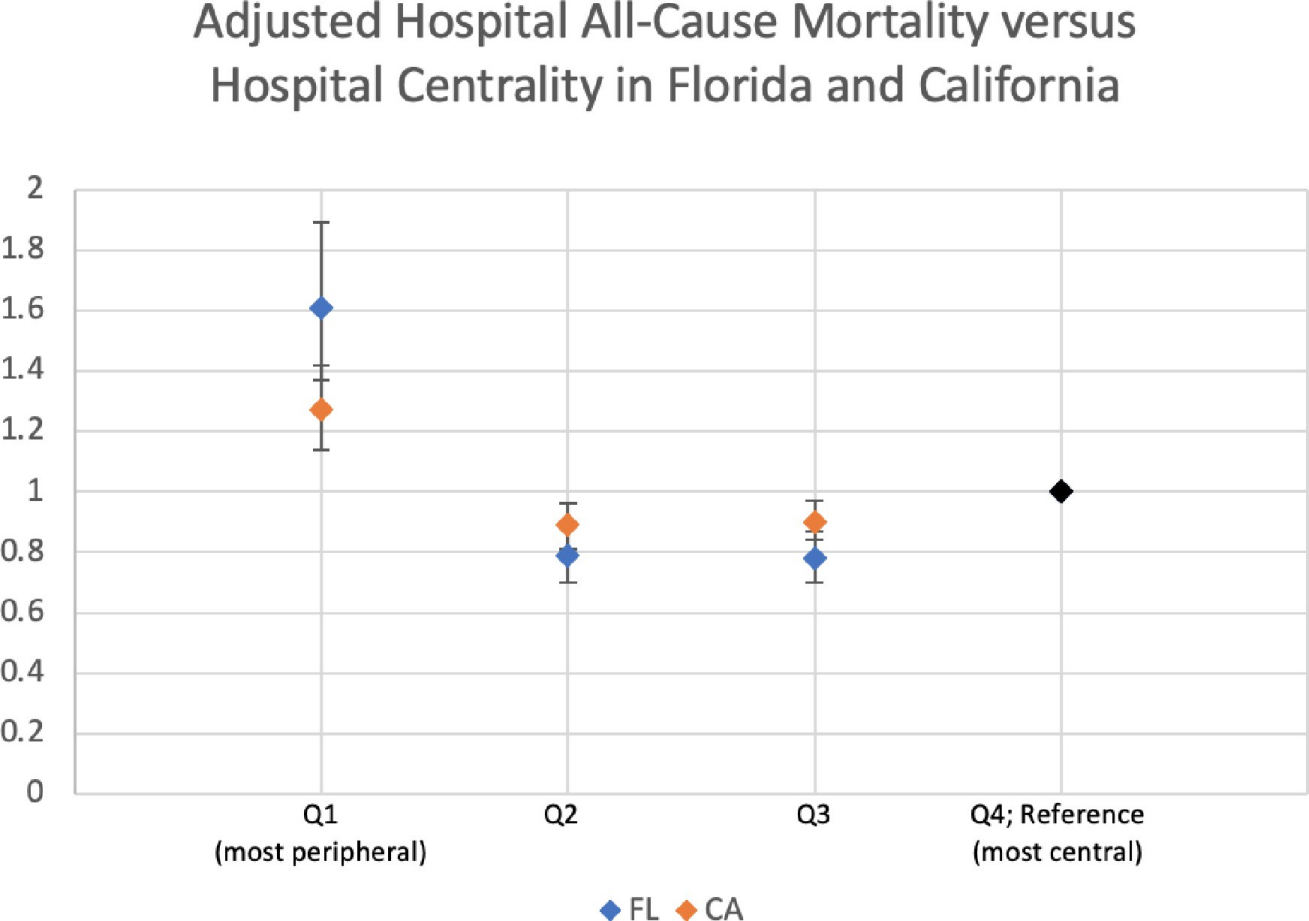
Adjusted hospital all–cause mortality versus hospital centrality in Florida and California.

We determined the association of hospital centrality quartile with all-cause and disease-related mortality rates and mean LOS with adjustment for patient and hospital characteristics (Table 2). Central hospitals (Q4) represent the reference category for the adjusted odds ratios. There was a U-shaped relationship between hospital centrality and inpatient mortality (Fig 4).

**Table 2:** Overall and disease–specific adjusted mortality and length of stay by hospital centrality quartile for Florida and California.

	All		Ischemic stroke		Heart Failure		Myocardial Infarction		Pneumonia	
	OR or IRR; (95%CI)	P-value	OR or IRR; (95%CI)	P-value	OR or IRR; (95%CI)	P-value	OR or IRR; (95%CI)	P-value	OR or IRR; (95%CI)	P-value
**FLORIDA**	**n = 1,246,169**		**n = 37,066**		**n = 178,573**		**n = 53,476**		**n = 128,855**	
**Adjusted In-hospital Mortality (OR, 95% CI)**										
Centrality Quartile 1	1.61 (1.37–1.89)	***<0*.*0001***	1.37 (0.95–1.99)	0.096	2.1 (1.76–2.51)	***<0*.*0001***	1.33 (1.03–1.73)	***0*.*030***	1.70 (1.42–2.02)	***<0*.*0001***
Quartile 2	0.79 (0.70–0.89)	***<0*.*0001***	0.82 (0.65–1.03)	0.083	1.0 (0.87–1.15)	0.997	0.93 (0.80–1.09)	0.364	0.88 (0.76–1.02)	0.080
Quartile 3	0.78 (0.70–0.87)	***<0*.*0001***	0.84 (0.69–1.02)	0.079	0.89 (0.79–0.998)	***0*.*050***	0.88 (0.77–1.00)	0.055	0.82 (0.72–0.93)	***0*.*002***
Quartile 4	Reference		Reference		Reference		Reference		Reference	
**Adjusted Length of Stay (IRR, 95% CI)**										
Centrality Quartile 1	2.47 (2.44–2.50)	***<0*.*0001***	1.24 (1.16–1.32)	***<0*.*0001***	2.64 (2.58–2.71)	***<0*.*0001***	1.64 (1.56–1.73)	***<0*.*0001***	2.65 (2.58–2.72)	***<0*.*0001***
Quartile 2	1.01 (1.00–1.02)	***<0*.*0001***	0.89 (0.86–0.92)	***<0*.*0001***	0.92 (0.91–0.93)	***<0*.*0001***	0.90 (0.88–0.92)	***<0*.*0001***	0.90 (0.89–0.91)	***<0*.*0001***
Quartile 3	0.95 (0.94–0.95)	***<0*.*0001***	0.92 (0.89–0.93)	***<0*.*0001***	0.96 (0.95–0.97)	***<0*.*0001***	0.96 (0.94–0.97	***<0*.*0001***	0.93 (0.92–0.94)	***<0*.*0001***
Quartile 4	Reference		Reference		Reference		Reference		Reference	
**CALIFORNIA**	**n = 1,415,728**		**n = 44,433**		**n = 215,807**		**n = 61,092**		**n = 164,976**	
**Adjusted In-hospital Mortality (OR, 95% CI)**										
Centrality Quartile 1	1.27 (1.14–1.42)	***<0*.*0001***	0.86 (0.62–1.18)	0.348	1.24 (1.07–1.44)	***0*.*0036***	1.53 (1.21–1.93)	***0*.*0001***	0.91 (0.79–1.04)	0.167
Quartile 2	0.89 (0.81–0.96)	***0*.*0046***	0.88 (0.74–1.05)	0.148	0.98 (0.88–1.08)	0.623	1.00 (0.88–1.14)	0.988	0.83 (0.75–0.91)	***<0*.*0001***
Quartile 3	0.90 (0.84–0.97	***0*.*008***	0.83 (0.72–0.95)	***0*.*008***	0.98 (0.90–1.07)	0.694	0.93 (0.83–1.03)	0.151	0.93 (0.86–1.01)	0.103
Quartile 4	Reference		Reference		Reference		Reference		Reference	
**Adjusted Length of Stay (IRR, 95% CI)**										
Centrality Quartile 1	1.44 (1.42–1.45)	***<0*.*0001***	1.11 (1.04–1.19)	***0*.*001***	1.17 (1.14–1.20)	***<0*.*0001***	1.48 (1.39–1.58)	***<0*.*0001***	1.02 (0.99–1.05)	0.184
Quartile 2	1.09 (1.08–1.09)	***<0*.*0001***	0.90 (0.88–0.93)	***<0*.*0001***	0.96 (0.94–0.97)	***<0*.*0001***	0.92 (0.90–0.94)	***<0*.*0001***	0.92 (0.90–0.93)	***<0*.*0001***
Quartile 3	0.98 (0.98–0.99)	***<0*.*0001***	0.97 (0.95–0.99)	***0*.*003***	0.96 (0.965–0.97)	***<0*.*0001***	0.93 (0.91–0.94)	***<0*.*0001***	0.99 (0.98–0.995)	***0*.*006***
Quartile 4	Reference		Reference		Reference		Reference		Reference	

### All-cause inpatient mortality

In Florida, compared to central hospitals where mortality was 1.60%, patients admitted to hospitals in the most peripheral hospitals had higher all-cause mortality (1.97%, adjusted OR (95%CI): Q1 1.61 (1.37, 1.89), p<0.0001). Also compared to central hospitals, patients admitted to hospitals in the intermediate quartiles had lower in-hospital all-cause mortality (%, adjusted OR (95% CI): Q2 1.39%, 0.79 (0.70, 0.89), p<0.001; Q3 1.33%, 0.78 (0.70, 0.87), p<0.0001). Patients at the most peripheral hospitals had longer lengths of stay (adjusted IRR (95% CI): Q1 2.47 (2.44, 2.50), p<0.0001). Patients admitted to hospitals in the intermediate quartiles had statistically significant but not meaningfully longer LOS relative to patients in the most central quartile (IRR (95% CI): Q2 1.01 (1.00, 1.02), Q3 0.95 (0.94–0.95), p<0.0001).

In California, all findings were consistent. Compared to central hospitals in which mortality was 2.16%, patients admitted to hospitals in the most peripheral hospitals had higher all-cause mortality (2.52%, adjusted OR (95% CI): Q1 1.27 (1.14–1.42). p<0.0001). The intermediate quartiles had lower all-cause mortality compared to central hospitals (% and adjusted OR (95% CI): Q2 2.08%, 0.89 (0.81–0.96), p = 0.0046; Q3 2.17%, 0.90 (0.84–0.97), p = 0.008). The LOS was also longer in both Q1 and Q2 peripheral hospitals (adjusted IRR (95% CI): Q1 1.44 (1.42–1.45), p<0.0001; Q2 1.09 (1.08–1.09), p<0.0001), and shorter in Q3 (adjusted IRR (95% CI): Q3 0.98 (0.98–0.99), p<0.0001).

### Ischemic stroke

In Florida, there were 44,433 discharges to 230 hospitals for ischemic stroke. The prior trends occurred for ischemic stroke-specific mortality as well. Relative to central hospitals, patients at hospitals in the most peripheral quartile had higher odds of death, and patients at hospitals in the intermediate quartiles had lower odds of death, though these findings were insignificant. Adjusted IRRs for length of stay were significantly different for each quartile relative to the most central quartile, where patients at hospitals in the most peripheral quartile had longer lengths of stay and patients in hospitals in the intermediate quartiles had shorter lengths of stay (IRR (95% CI): Q1 1.24 (1.16–1.32); Q2 0.89 (0.86–0.92); Q3 0.92 (0.89–0.93), all p<0.0001; Q4 (reference)). The findings were consistent in California, as well. In California, the mortality analysis showed that quartiles 1 and 2 were not significantly different from reference, whereas quartile 3 showed significantly lower mortality than the most central quartile.

### Heart failure

In Florida, there were 178,573 discharges to 257 hospitals for heart failure. The prior trends were partially replicated for heart failure-specific mortality. Relative to central hospitals, patients at hospitals in the most peripheral quartile had significantly higher odds of death, but patients at hospitals in quartile 2 were no different from the reference, and those in quartile 3 were borderline significant for reduced mortality (OR (95% CI): Q1 2.10 (1.76–2.51), p<0.0001; Q2 1.00 (0.87–1.15), p = 0.997; Q3 0.89 (0.79–1.00), p = 0.050; Q4 (reference)). For LOS, patients at hospitals in the most peripheral quartile had longer lengths of stay and patients in hospitals in the intermediate quartiles had shorter lengths of stay (IRR (95% CI): Q1 2.64 (2.58–2.71), p<0.0001; Q2 0.92 (0.91–0.93), p<0.0001; Q3 0.96 (0.95–0.97), p<0.0001; Q4 (reference)). All findings were consistent in California except for quartile 3 of the mortality analysis, which showed no significant difference from the most central quartile.

### Acute myocardial infarction

In Florida, there were 53,476 discharges for acute myocardial infarction identified in 233 hospitals. The prior trends were partially replicated for acute myocardial infarction-specific mortality. Relative to central hospitals, patients at hospitals in the most peripheral quartile had significantly higher odds of death, but patients at hospitals in the intermediate quartiles did not have significantly different odds of death (OR (95% CI): Q1 1.33 (1.03–1.73), p = 0.030; Q4 (reference)). For LOS, patients at hospitals in the most peripheral quartile had longer lengths of stay and patients in hospitals in the intermediate quartiles had shorter lengths of stay (IRR (95% CI): Q1 1.64 (1.56–1.73), p<0.0001; Q2 0.90 (0.88–0.92), p<0.0001; Q3 0.96 (0.94–0.97), p<0.0001; Q4 (reference)). All findings were similar in California with significantly worse outcomes in peripheral hospitals.

### Pneumonia

In Florida, there were 128,855 discharges to 257 hospitals for pneumonia. The prior trends were replicated for pneumonia-specific mortality. Relative to central hospitals, patients at hospitals in the most peripheral quartile had significantly higher odds of death. Patients at the second most peripheral quartile did not have significantly different odds of death, but patients at the third most central quartile did have significantly lower odds of death (OR (95% CI): Q1 1.70 (1.42–2.02), p<0.0001; Q2 0.88 (0.76–1.02), p = 0.080; Q3 0.82 (0.72–0.93), p = 0.002; Q4 (reference)). For LOS, patients at hospitals in the most peripheral quartile had longer lengths of stay and patients in hospitals in the intermediate quartiles had shorter lengths of stay (IRR (95% CI): Q1 2.65 (2.58–2.72), p<0.0001; Q2 0.90 (0.89–0.91), p<0.0001; Q3 0.93 (0.92–0.94), p<0.0001; Q4 (reference)). In California, there was only partial replication of the prior trends. For inpatient mortality, peripheral hospitals were not found to have statistically different findings, but the intermediate hospital did have significantly better outcomes. For LOS, there was a similar pattern with peripheral hospital no worse, and intermediate hospitals better than central hospitals.

## Discussion

In this exploratory analysis of two large state-based hospital networks based on patient-sharing between hospitals, we found evidence that factors emerging from the network, beyond traits of individual patients or hospitals, may strain peripheral and central hospitals. Within two large US state-based hospital networks, the most peripheral and the most central hospitals had higher mortality and longer lengths of stay, while hospitals of intermediate centrality had lower mortality and shorter lengths of stay. These findings persist after adjusting for common patient and hospital risk factors, and within specific high acuity illnesses, although are preliminary and need validation in other data sets with more detailed clinical data. Further investigations could also examine data from other countries for international generalizability. This U-shaped relationship (Fig 4) supports the interdependence of hospitals. Further investigation with in-depth clinical data is warranted to further account for illness severity and patient complexity to disentangle network and patient-related effects.

Supported by the network vulnerability literature [26,27], we theorize that peripheral hospitals are subject to ‘low resource strain’ and central hospitals are subject to ‘over-reliance strain’, due to their respective positions in the network. In other words, peripheral hospitals are vulnerable to poorer outcomes due to a lack of access to resources or specialized care afforded to more connected facilities. Central hospitals, on the other hand, are vulnerable perhaps due to over-reliance within the network that strains their capacity for caring for complex patients. More than half of hospitalizations occurred in hospitals in the most central quartile. Central hospitals also care for complex patients whose degree of medical complexity cannot be fully accounted for in the analysis. In oncology, for example, more central hospitals can have more fragmented care due to the number and complexity of patients [28,29]. Accordingly, the intermediately central hospitals are buffered from both of these types of strain; they can off-load complex cases to their more central neighbors, while maintaining enough resources for the average patient. Thoughtful centralization may be able to mitigate these challenges, as centralization within hospital network structures has also been associated with better outcomes [30].

This study also contributes to the wider literature examining the role of physician- and hospital-based performance networks on measures of quality, safety, outcomes, and costs. Escalating costs and variable patient outcomes have driven intense interest in comparative benchmarking and value-based health care efforts [31,32]. These initiatives range from reports such as US News and World Report rankings to pay-for-performance, bundled payments or Alternative Quality Contracts. Benchmarking relies to some degree on accurate comparison of costs and/or outcomes by physician or facility [33]. However, these efforts currently neglect the interconnected nature of hospitals and may benefit from greater consideration of interhospital network effects.

This study must be interpreted in the context of its limitations. First, as an observational study, this work carries the limitations of other non-randomized studies, including the possibility of confounding. We attempted to control for patient factors through use of the CCI, but other acute or chronic conditions and unmeasured factors may confound results. Additionally, comorbid diseases may interact, leading to elevated complexity beyond the additive nature of the CCI. These limitations may lead to under-reporting of patient complexity, which may be particularly pertinent for complex patients at central hospitals, leading to under-adjustment of their risk. As stated above, future analyses should be conducted in richer clinical datasets to validate the findings of this study. Second, our attempt to control for hospital-related factors is limited by collinearity between central hospitals and large hospitals. Third, the HCUP SID datasets include all payers for all inpatient care obtained within the state but does not include information for patients who receive care in multiple states. Therefore, the networks we have created are restricted to patient sharing between hospitals within the state. Nonetheless, we expect that the number of patients who have been hospitalized in Florida and California and are then hospitalized in a different state would be limited. Fourth, these two state-specific networks may not generalize to other states or the US as a whole, though we did include states with large and heterogenous populations (~18% of the US population). Further work should include states with differing populations, locations, and healthcare system organization, as well as newer datasets or datasets from other countries. Fifth, these networks do not represent patient-sharing across all settings of healthcare use, but rather, only inpatient and emergency department utilization. Future analyses would benefit from inclusion of ambulatory settings, post-acute care settings such as skilled nursing facilities, and multi-year longitudinal data. Accounting for sharing of staff and physicians may also be helpful. Finally, from a network perspective, we focused on weighted degree which reflects patient sharing between hospitals based on number of shared patients. Future work could investigate the network topology with additional metrics.

## Conclusion

Hospitals are interdependent based on patient-sharing. Our results show that the position of hospitals within an inter-hospital network is associated with patient outcomes. Specifically, hospitals located in the peripheral or central positions may be most vulnerable to diminished quality outcomes. While it is intuitively obvious that patients often use healthcare from different hospitals or settings of care, this study further demonstrates that this interconnectivity is associated with patient outcomes. With further validation with deep clinical data, network properties should be considered in the accurate assessment of hospital performance. Future network studies with clinical datasets may be able to elucidate areas of weakness in the network which may be targets for quality improvement interventions.

## Supporting information

S1 Appendix (DOCX)

S2 Appendix (DOCX)

S3 Appendix (DOCX)

S4 Appendix (DOCX)

S5 Appendix (DOCX)

S6 Appendix (DOCX)
