# Supplementary material for: Association of hospital centrality in inter-hospital patient-sharing networks with patient mortality and length of stay
Source: PLoS One. 2023 Mar 15;18(3):e0281871. doi: 10.1371/journal.pone.0281871 (PMC10016671; doi:10.1371/journal.pone.0281871)
Supplement: S1 Appendix — (DOCX) [file pone.0281871.s001.docx]

Appendix 1. Hospital characteristics For Florida and California

|  | **Florida** | |  | **California** | |
| --- | --- | --- | --- | --- | --- |
|  | N | % |  | N | % |
| **Hospitals** | 272 | 100.0 |  | 396 | 100.0 |
| **Control ownership of hospital** |  |  |  |  |  |
| Private, non-for-profit | 109 | 40.1 |  | 219 | 55.3 |
| Government, nonfederal | 28 | 10.3 |  | 72 | 18.2 |
| Investor-owned, for profit | 135 | 49.6 |  | 105 | 26.5 |
| **Teaching status** |  |  |  |  |  |
| Major Teaching | 23 | 8.5 |  | 20 | 5.1 |
| Minor Teaching | 58 | 21.3 |  | 77 | 19.4 |
| Non-teaching | 191 | 70.2 |  | 299 | 75.5 |
| **Hospital bed-size** |  |  |  |  |  |
| 1-99 beds | 88 | 32.4 |  | 124 | 31.3 |
| 100-199 beds | 64 | 23.5 |  | 113 | 28.5 |
| 200-299 beds | 41 | 15.1 |  | 65 | 16.4 |
| >= 300 beds | 79 | 29.0 |  | 94 | 23.7 |
