# Supplementary material for: Association of hospital centrality in inter-hospital patient-sharing networks with patient mortality and length of stay
Source: PLoS One. 2023 Mar 15;18(3):e0281871. doi: 10.1371/journal.pone.0281871 (PMC10016671; doi:10.1371/journal.pone.0281871)
Supplement: S3 Appendix — (DOCX) [file pone.0281871.s003.docx]

Appendix 3: California Patient and Hospital Characteristics by Quartile of Hospital Network Centrality
