# Supplementary material for: Association of hospital centrality in inter-hospital patient-sharing networks with patient mortality and length of stay
Source: PLoS One. 2023 Mar 15;18(3):e0281871. doi: 10.1371/journal.pone.0281871 (PMC10016671; doi:10.1371/journal.pone.0281871)
Supplement: S4 Appendix — (DOCX) [file pone.0281871.s004.docx]

Appendix 4: Overall and disease-specific unadjusted and adjusted mortality and length of stay by hospital centrality quartile for Florida

*Patient characteristics included Age, Gender, Race, Median household, income (Q1, Q2, Q3, Q4), Patient location (Large metropolitan area, small metropolitan area, micropolitan area), Payer (Medicare, private insurance, Medicaid, Self-pay, other) , Charlson comorbidities (0, 1, 2, 3, >3). Hospital characteristics included Control ownership of hospital (Private, Government, Investor-owned) , Teaching status, Hospital bedside (1-99 beds, 100-199 beds, 200-299 beds, >=300 beds) .

** IRR - Incident rate ratio
