# Supplementary material for: Association of hospital centrality in inter-hospital patient-sharing networks with patient mortality and length of stay
Source: PLoS One. 2023 Mar 15;18(3):e0281871. doi: 10.1371/journal.pone.0281871 (PMC10016671; doi:10.1371/journal.pone.0281871)
Supplement: S6 Appendix — (DOCX) [file pone.0281871.s006.docx]

Appendix 6. ICD-9-CM diagnosis codes used to identify condition-specific patient cohorts

|  | ICD-9-CM Diagnosis Code | Long Descriptions |
| --- | --- | --- |
| Acute MI |  |  |
|  | 410.00 | Acute Myocardial Infarction (anterolateral wall) – episode of care unspecified |
|  | 410.01 | Acute Myocardial Infarction (anterolateral wall) – initial episode of care |
|  | 410.10 | Acute Myocardial Infarction (other anterior wall) – episode of care unspecified |
|  | 410.11 | Acute Myocardial Infarction (other anterior wall) – initial episode of care |
|  | 410.20 | Acute Myocardial Infarction (inferolateral wall) – episode of care unspecified |
|  | 410.21 | Acute Myocardial Infarction (inferolateral wall) – initial episode of care |
|  | 410.30 | Acute Myocardial Infarction (inferoposterior wall) – episode of care unspecified |
|  | 410.31 | Acute Myocardial Infarction (inferoposterior wall) – initial episode of care |
|  | 410.40 | Acute Myocardial Infarction (other inferior wall) – episode of care unspecified |
|  | 410.41 | Acute Myocardial Infarction (other inferior wall) – initial episode of care |
|  | 410.50 | Acute Myocardial Infarction (other lateral wall) – episode of care unspecified |
|  | 410.51 | Acute Myocardial Infarction (other lateral wall) – initial episode of care |
|  | 410.60 | Acute Myocardial Infarction (true posterior wall) – episode of care unspecified |
|  | 410.61 | Acute Myocardial Infarction (true posterior wall) – initial episode of care |
|  | 410.70 | Acute Myocardial Infarction (subendocardial) – episode of care unspecified |
|  | 410.71 | Acute Myocardial Infarction (subendocardial) – initial episode of care |
|  | 410.80 | Acute Myocardial Infarction (other specified site) – episode of care unspecified |
|  | 410.81 | Acute Myocardial Infarction (other specified site) – initial episode of care |
|  | 410.90 | Acute Myocardial Infarction (unspecified site) – episode of care unspecified |
|  | 410.91 | Acute Myocardial Infarction (unspecified site) – initial episode of care |
|  |  |  |
| Heart Failure |  |  |
|  | 402.01 | Malignant hypertensive heart disease with heart failure |
|  | 402.11 | Benign hypertensive heart disease with heart failure |
|  | 402.91 | Hypertensive heart disease with heart failure |
|  | 404.01 | Malignant hypertensive heart and renal disease with heart failure |
|  | 404.03 | Malignant hypertensive heart and renal disease with heart failure and renal failure |
|  | 404.11 | Benign hypertensive heart and renal disease with heart failure |
|  | 404.13 | Benign hypertensive heart and renal disease with heart failure and renal failure |
|  | 404.91 | Hypertensive heart and renal disease with heart failure |
|  | 404.93 | Hypertensive heart and renal disease with heart failure and renal failure |
|  | 428.0 | Congestive heart failure |
|  | 428.1 | Left heart failure |
|  | 428.20 | Unspecified systolic heart failure |
|  | 428.21 | Acute systolic heart failure |
|  | 428.22 | Chronic systolic heart failure |
|  | 428.23 | Acute on chronic systolic heart failure |
|  | 428.30 | Unspecified diastolic heart failure |
|  | 428.31 | Acute diastolic heart failure |
|  | 428.32 | Chronic diastolic heart failure |
|  | 428.33 | Acute on chronic diastolic heart failure |
|  | 428.40 | Unspecified combined systolic and diastolic heart failure |
|  | 428.41 | Acute combined systolic and diastolic heart failure |
|  | 428.42 | Chronic combined systolic and diastolic heart failure |
|  | 428.43 | Acute on chronic combined systolic and diastolic heart failure |
|  | 428.9 | Heart failure, unspecified |
|  |  |  |
| Pneumonia |  |  |
|  | 481 | Pneumococcal pneumonia (Streptococcus pneumoniae pneumonia) |
|  | 482.0 | Other bacterial pneumonia: pneumonia due to Klebsiella pneumoniae |
|  | 482.1 | Other bacterial pneumonia: pneumonia due to Pseudomonas |
|  | 482.2 | Other bacterial pneumonia: pneumonia due to Hemophilus influenzae (H. influenzae) |
|  | 482.30 | Pneumonia due to Streptococcus: Streptococcus, unspecified |
|  | 482.31 | Pneumonia due to Streptococcus: Group A |
|  | 482.32 | Pneumonia due to Streptococcus: Group B |
|  | 482.39 | Pneumonia due to Streptococcus: Other Streptococcus |
|  | 482.40 | Pneumonia due to Staphylococcus, unspecified |
|  | 482.41 | Methicillin susceptible pneumonia due to Staphylococcus aureus |
|  | 482.42 | Methicillin resistant pneumonia due to Staphlococcus aureus |
|  | 482.49 | Pneumonia due to other Staphylococcus pneumonia |
|  | 482.82 | Pneumonia due to Escherichia coli (E. coli) |
|  | 482.83 | Pneumonia due to other gram-negative bacteria |
|  | 482.84 | Pneumonia due to Legionnaires’ disease |
|  | 482.89 | Pneumonia due to other specified bacteria |
|  | 482.9 | Bacterial pneumonia unspecified |
|  | 483.0 | Pneumonia due to Mycoplasma pneumoniae |
|  | 483.1 | Pneumonia due to Chlamydia |
|  | 483.8 | Pneumonia due to other specified organism |
|  | 485 | Bronchopneumonia, organism unspecified |
|  | 486 | Pneumonia, organism unspecified |
|  |  |  |
| Ischemic Stroke |  |  |
|  | 433.01 | Occlusion and stenosis of basilar artery with cerebral infarction |
|  | 433.11 | Occlusion and stenosis of carotid artery with cerebral infarction |
|  | 433.21 | Occlusion and stenosis of vertebral artery with cerebral infarction |
|  | 433.31 | Occlusion and stenosis of multiple and bilateral precerebral arteries with cerebral infarction |
|  | 433.81 | Occlusion and stenosis of other specified precerebral artery with cerebral infarction |
|  | 433.91 | Occlusion and stenosis of unspecified precerebral artery with cerebral infarction |
|  | 434.01 | Cerebral thrombosis with cerebral infarction |
|  | 434.11 | Cerebral embolism with cerebral infarction |
|  | 434.91 | Cerebral artery occlusion, unspecified with cerebral infarction |
|  | 436 | Acute, but ill-defined, cerebrovascular disease |
